# Response of Palladium and Carbon Nanotube Composite Films to Hydrogen Gas and Behavior of Conductive Carriers

**DOI:** 10.3390/ma13204568

**Published:** 2020-10-14

**Authors:** Muxuan Zou, Yoshinori Aono, Shuhei Inoue, Yukihiko Matsumura

**Affiliations:** 1 Department of Mechanical Science and Engineering, Hiroshima University, 1-4-1 Kagamiyama, Higashi-Hiroshima 739-8527, Japan; m180602@hiroshima-u.ac.jp; 2 Mechanical Engineering Program, Graduate School of Advanced Science and Engineering, Hiroshima University, 1-4-1 Kagamiyama, Higashi-Hiroshima 739-8527, Japan; m205580@hiroshima-u.ac.jp (Y.A.); mat@hiroshima-u.ac.jp (Y.M.)

**Keywords:** resistive sensors, carbon nanotube, hydrogen, sensor, DFT, thin film

## Abstract

To develop a high-performance hydrogen gas sensor, we fabricated a composite film made of carbon nanotubes (CNTs) and palladium nanoparticles. Carbon nanotubes were spin-coated onto a glass substrate, and subsequently, palladium nanoparticles were sputtered onto this film. The response to hydrogen gas was measured during two seasons (summer and winter) using a vacuum chamber by introducing a hydrogen/argon gas mixture. There was a clear difference in the sensor response despite the temperature difference between summer and winter. In addition, since a clean chamber was used, fewer water molecules acted as a dopant, and the behavior of the CNT changed from p-type to n-type because of the dissociative adsorption of hydrogen. This phenomenon was confirmed as the Seebeck effect. Finally, the work functions of Pd, PdH_x_, and CNT were calculated by first-principle calculations. As predicted by previous studies, a decrease in work function due to hydrogen adsorption was confirmed; however, the electron transfer to CNT was not appropriate from the perspective of charge neutrality and was found to be localized at the Pd/CNT interface. It seems that the Seebeck effect causes the concentration of conductive carriers to change.

## 1. Introduction

Hydrogen gas is extensively used in aircraft, vehicles, fuel cells, and as a clean source of energy [1]. However, hydrogen gas can react with oxygen, and the gas mixtures can spontaneously explode when the concentration of H2 gas in the air is between 4% and 75.6%. As a result, during hydrogen production, utilization, and transportation, hydrogen safety equipment and sensing materials must be considered [2,3]. Although the calorific value per unit weight of H2 is extremely high, it is only one-third of that of natural gas. Hence, H2 is often stored in high-pressure tanks or metal hydride storage systems and is usually utilized as liquid hydrogen [4,5]. In this context, several previous studies have reported extensive investigation of hydrogen-sensitive materials, such as palladium (Pd) [6,7,8,9], for better hydrogen sensing or storage.

Conventionally, semiconductor materials were used to fabricate hydrogen sensors. However, the working temperature of these sensors is usually around 400 °C. Even though its working temperature is below the flashpoint, hydrogen is highly flammable and explosive and yields a far-from-ideal sensor. Undoubtedly, to drive the sensor, we must prepare a power supply system; but this may be problematic when used for other applications. Furthermore, semiconductor materials such as SnO2, ZnO can react with other gases like methanol and ethanol at such a high temperature. Thus, it is a matter of gas selectivity [10]. With the rapid development and progress of nanotechnology, the focus of palladium-based electronic hydrogen sensors has turned to nanostructure-based sensors. Referring to previous studies, palladium-based hydrogen sensors can be categorized into three types: palladium thin-film hydrogen sensors, palladium nanowires, and palladium nanoclusters. For pure palladium hydrogen sensor, it was often found that inaccuracy and hysteresis in the measurement, and palladium film was usually damaged because of palladium volume expansion induced by hydrogen absorption [11,12,13]. Palladium nanowire [14,15,16,17,18,19] hydrogen sensors have exhibited impressive characteristics such as fast hydrogen response and good repeatability. However, precise diameter control and preparation are still a challenge. In case of palladium nanocluster structures [20,21], previous studies have employed single-walled carbon nanotubes (SWCNTs) modified with dendrimers. Despite their excellent performance, complicated preparation procedures may prevent many potential applications.

In the development of high-performance hydrogen sensors, sensors based on Pd and CNT (carbon nanotube) composite materials have attracted significant attention because of their advantages of hydrogen selectivity, high sensitivity [22], real-time detection, and low power consumption. CNTs coated with Pd nanoparticles [23,24] are suitable for their ease of fabrication and mass production; consequently, hydrogen sensors are in high demand.

For resistance changes occurring after hydrogen adsorption in the case of CNTs/Pd hydrogen sensors, studies have offered multiple reasons. One possibility is that CNTs work as a highway for electron transportation. After hydrogen adsorption, the work function of PdH_x_ becomes lower than that of Pd. Thus, PdH_x_ formation allows more electrons to be transferred from Pd nanoparticles to the CNTs. Electrons would now combine with the hole carriers in the CNTs, decreasing the number of carriers and increasing the electric resistance of the sensor [25]. However, another study points out that the Pd nanocluster structures can also influence the resistance of the sensors. After the volume of PdH_x_ expands, Pd nanoparticles come in contact more closely and tightly, which can improve their conductivity [26].

In this study, we propose a new method of fabrication for hydrogen sensors composed of multi-walled carbon nanotube (MWCNT) films coated with Pd nanoparticles that exhibit high sensor performance as well as repeatability and are suitable for mass production. Subsequently, we have also focused on their response behavior. Contrary to previous reports, we observed a very interesting phenomenon of switching of major conductive carriers by investigating the response to hydrogen in a vacuum chamber.

## 2. Materials and Methods

### 2.1. Preparation of the Pd/CNT Film

**Figure 1:**
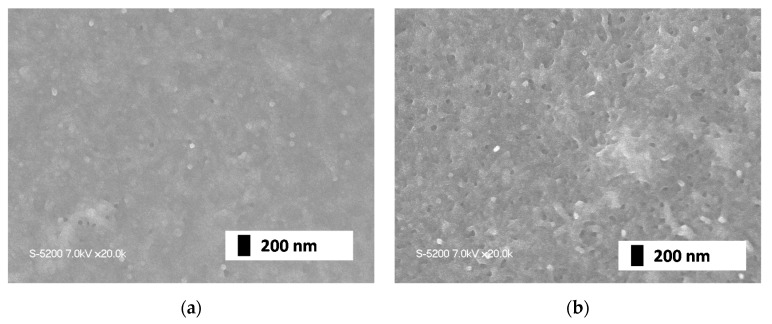
Surface morphologies. (**a**) SEM image of Sample 2.13-1(**b**) SEM image of Sample 2.13-2

**Figure 2:**
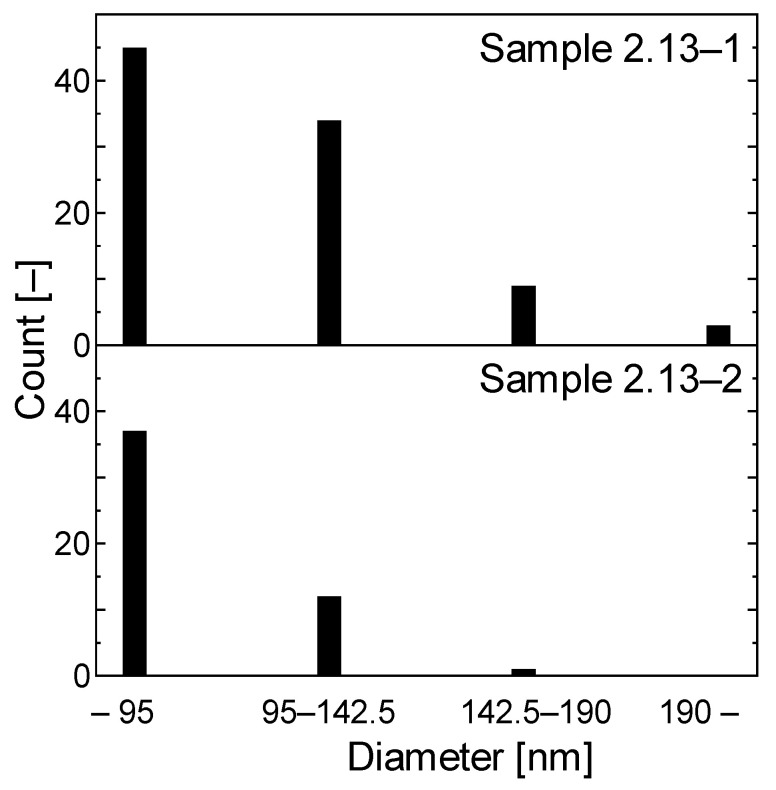
Histogram of palladium nano particles.

First, we fabricated CNT thin-films using an MWCNT solution (Meijio Nano Carbon Co. Ltd. CP163CP, Nagoya, Japan) on a 25 mm square glass substrate by the spin-coating method. We controlled the thickness of the film through the number of spin coatings; then, we measured transmittance of light of 632 nm to roughly check the thickness and make the thickness uniform for each samples. Under the centrifugal force, the CNT solution spreads onto the surface of the substrate uniformly and smoothly. After spin coating, the substrate was placed in an oven at 40 °C to completely remove the moisture. Afterward, we used a sputtering system to deposit the Pd nanoparticles onto the CNT film. We employed magnetron sputtering source of Kurt J. Lesker. A sputtering target is pure palladium (higher 99.5%) disk of 1 inch in diameter. We applied DC 500 V (input power was 6 W) and argon was introduced of 20 sccm at 10 Pa. We controlled the amount of Pd nanoparticles deposited by setting the sputtering time, and we prepared two samples of which synthesis conditions are summarized in Table 1. Figure 1a,b indicate surface morphologies of Sample 2.13-1 and 2.13-2, respectively, observed by scanning electron microscope (Hitachi High Technology, S-5200), and Figure 2 indicates their particle distributions and concentrations. We counted number of particles in the area of 130 μm^2^. Comparing the two images, there is not much difference in the size of the nanoparticles, and the number increases with the sputtering time. This is because the amount of sputtered palladium was too small to aggregate on the surface.

### 2.2. Measurement of Sensor Response

**Figure 3:**
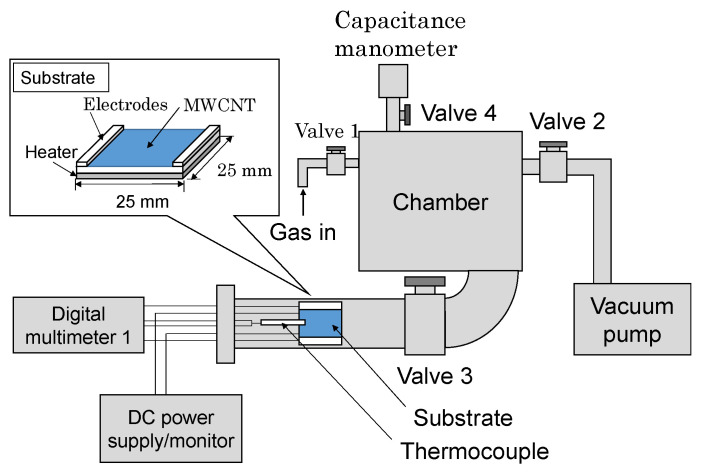
Experimental setup of the measurement system.

**Figure 4:**
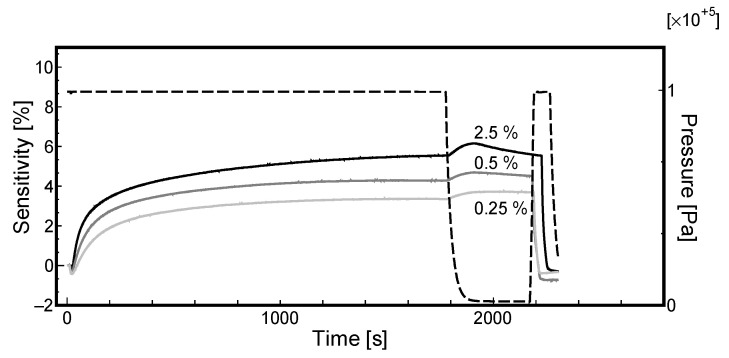
Effect of sensitivity of Sample 213-2 on hydrogen concentrations.

**Figure 5:**
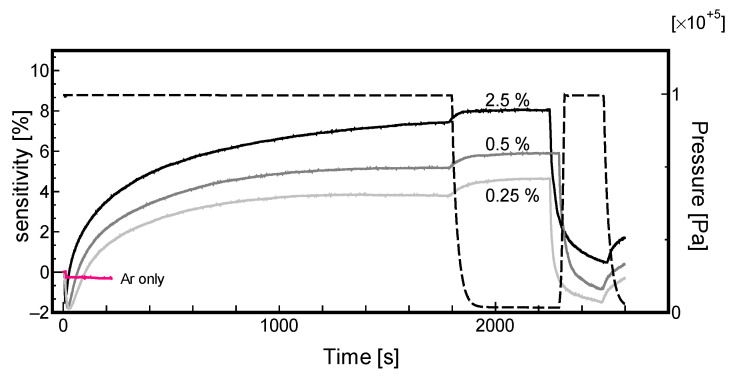
Effect of sensitivity of Sample 213-1 on hydrogen concentrations.

**Figure 6:**
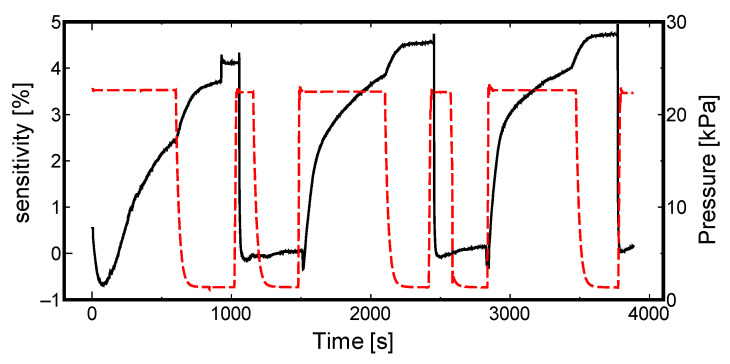
Sample 213-1 winter (less than 10 °C) sensitivity measurement.

**Figure 7:**
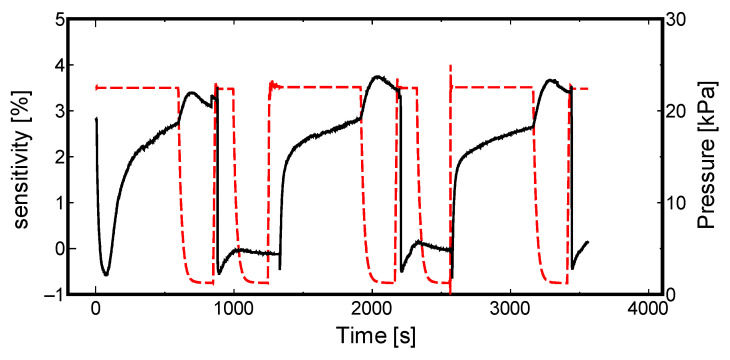
Sample 213-1 summer (more than 25 °C) sensitivity measurement.

Figure 3 presents a schematic of our measurement system. The measurement section (main chamber) consists of stainless chamber (ICF070 defined by ISO), which is directly connected to a buffer chamber that is larger than 200 times in volume. The substrate coated with Pd/CNT nanoparticles was placed in the alumina casing fitted with a heater and a thermocouple; a digital multimeter and DC power supply were connected via feedthrough in order to measure the sheet resistance of CNT film. A typical procedure is as follows: First, the main and the buffer chambers were evacuated by a rotary vacuum pump to approximately 10 Pa; subsequently, valves 1 and 2 were closed and valve 3 was opened to introduce Ar/H_2_ (5 vol.% of H_2_) gas up to the target pressure (1 × 10^5^ Pa for Figure 4 and Figure 5, 0.25 × 10^5^ Pa for Figure 6 and Figure 7). Finally, we closed valve 3 and opened valve 2 for the measurement. If we anneal the substrate, this procedure should be conducted under vacuum at 60 °C. After the experiment, we opened valve 4 and introduced oxygen to remove hydrogen from the sample.

### 2.3. Simulation

The calculations were carried out using the Atomistix ToolKit-Virtual Nano Laboratory (ATK-VNL) first principle calculation simulation software of the DFT-based package. From the calculations, local density approximation (LDA) and generalized gradient approximation (GGA) were used to estimate the work function to discuss the electron transfer from hydrogen to the MWCNT sensor. Models for the (100), (110), and (111) surfaces of Pd and PdH_x_ were constructed, and the work functions were calculated. For simplicity, MWCNTs are expressed as double-walled carbon nanotubes (DWCNTs) of (12, 12)-(6, 6).

For this simulation, Perdew-Burke–Ernzerhof Exchange (PBE, for GGA) [27,28] and Perdew Zunger (PZ, for LDA) [29] were employed as functionals, while Perdew–Burke–Ernzerhof Correlation was used as an exchange-correlation function for GGA. Double zeta polarized (DZP, one confined orbital and one analytical split for each occupied valence orbital in the atom; one polarization orbital for the first unoccupied shell in the atom) for GGA and single zeta polarized (SZP, one confined orbital for each occupied valence orbital in the atom, and one polarization orbital for the first unoccupied shell in the atom) for LDA were applied as the basis sets.

## 3. Results and Discussion

We monitored the electric resistance of the Pd/CNT film. To express the sensor responsivity, we defined Equation (1):

(1) $$S=\frac{R_{0}-R}{R_{0}}$$

where *S* is the sensor responsivity, *R*_0_ is the resistance in the vacuum condition, and *R* indicates the resistance depending on the concentration of hydrogen.

Numerous experiments on this type of CNT-based sensor have been reported. We have established the mechanism of changes in the electric resistance during physical adsorption [30,31,32]. In brief, the electric resistance increases because the energy of the conduction electrons moving between CNTs connected in a spaghetti-like pattern increases with the gas concentration increases. As this change depends on the dielectric constant, the electric resistance will always increase. If the electric resistance decreases, it signifies an increase in the conductive carriers due to the chemical bonding between the gas molecules and CNTs.

First, we focused on the effect of the thickness of the Pd film. Figure 4 and Figure 5 display the sensor response for the samples 213-2 and 213-1, respectively, in the hydrogen gas concentration range of 2.5% to 0.25%. The results presented that a higher hydrogen gas concentration results in higher sensitivity. In these figures, the dashed lines indicate the pressure of the Ar/H_2_ mixture, while the three curved lines represent the sensitivity. As the sensitivity exhibited a positive value, as explained above, the number of conductive carriers increased. The plausible mechanism reported in related literature explained that the hydrogen molecules were dissociatively adsorbed onto the palladium nanoparticles, and the electrons were transferred into CNT. The reason is discussed later; however, the most notable thing here is that the electric resistance is decreasing unlike other literatures. Furthermore, it should be noted that the sensitivity becomes negative immediately after introducing hydrogen; that is, an increase in electric resistance is observed momentarily. This phenomenon is often observed for the first time even in case of the results of the repeated characteristics.

In both the experiments, the gas supply was stopped, and the sensitivity was found to increase slightly after evacuation with a vacuum pump (t = 1800–2000 s); however, the reason for this is not clear. Consequently, the gas pressure was found to rise, which indicated the pressure of oxygen introduced to remove the adsorbed hydrogen. As the amount of Pd in sample 213-2 is greater than that in sample 213-1, for every hydrogen concentration, the sensitivity of the sample 213-1 is higher than that of sample 213-2. The reason for this could be that the sensor follows Sieverts’ law [33] as described in Equation (2), which signifies that with an increase in Pd-H bonding points, the sensor can achieve higher sensitivity. Thus, the thicker Pd film shows higher sensitivity (a larger number of Pd particles).

(2) $$S=\frac{\left[H\right]}{\left[\mathrm{Pd}\right]}=\frac{1}{K_{S}}{\left(P_{H_{2}}\right)}^{0.5}$$

Second, we focused on the effect of temperature on the sensitivity of the sensor. As reported in our previous research [34], the adsorption equilibrium constant is affected by the temperature, which in turn mildly influences the response of the resulting sensor. Figure 6 and Figure 7 display the data measured in winter (temperature less than 10 °C) and summer (temperature more than 25 °C), respectively, using sample 213-1, which showed higher sensitivity in Figure 4 and Figure 5; the solid line illustrates how the sensor sensitivity changes, and the dashed line illustrates the main chamber pressure change.

Referring to these figures, the same sample, 213-1, takes a shorter time to reach its saturated state in summer (more than 25 °C). This is a matter of activation energy, according to previous research on the formation conditions and characteristics of metal hydrides [35]. In winter, slightly higher sensitivity is observed. Evidently, it alludes to the advantages of storing hydrogen at lower temperatures.

As we mentioned earlier, in previous studies [36], it is considered that palladium dissociates and adsorbs hydrogen; subsequently, electrons are transferred to the CNTs via palladium which changes the electric resistance of CNT thin films. Palladium adsorbs hydrogen and forms PdH_x_ at some point; however, the work function becomes smaller. The lower work function associated with PdHx formation leads to the transfer of more electrons from the Pd nanoparticles to the CNT layer. There are fewer carriers in CNTs so they can behave as both p- and n-type depending on the dopants. Usually, MWCNTs exhibit p-type behavior because of oxygen and/or water molecule dopants in the atmosphere. According to the results presented in previous studies, the electric resistance decreases, so that it is consistent with this common sense, because electrons donated from hydrogen must reduce the conductive carries of hole; however, herein MWCNTs exhibit n-type behavior, as described above.

**Figure 8:**
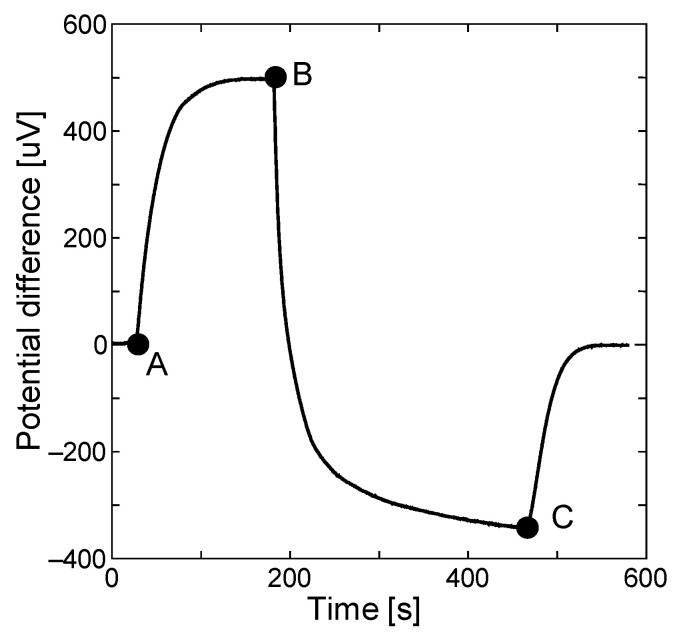
Seebeck effect.

To investigate this strange fact, we measured the Seebeck effect. We applied a temperature gradient in the Pd/CNT film. We placed the film in the chamber, cooled and subsequently, heated the left side. The potential difference was measured using the expression: *V*_L_–*V*_R_. First, we evacuated the chamber and commenced the cooling and heating (A in Figure 8). After 200 s, the potential difference appeared to be saturated (B). At this stage, since the potential difference is positive, the main conductive carrier is a hole which is p-type behavior. Second, we introduced hydrogen into the chamber (B); the potential difference suddenly decreased and finally became negative (C). This behavior indicated that our film changed from p-type to n-type. Thereafter, we stopped the cooling and heating processes, so that the potential difference became zero.

This is a very interesting phenomenon. It is considered that this kind of carrier conversion was observed because CNTs inherently possess only a few conductive carriers as it was reported that they could become both p- and n-type depending on the dopant. It is interesting to note why this phenomenon, in which electrons are indirectly transferred by adsorbing hydrogen and finally changing to n-type, has not been observed in previous experiments. A considerable amount of water may act as a dopant so that hydrogen adsorption cannot induce the conductive carrier switch. However, since we initially conducted experiments in the vacuum chamber, it exhibited p-type behavior, which subsequently changed to n-type behavior because of the conductive carrier switch.

Finally, we calculated the work functions of Pd, PdH_x_ (interstitial solid solution type), and DWCNT [(12, 12)-(6, 6)], which are originally MWCNTs. The results are summarized in Table 2. For Pd and PdH_x_, we calculated three crystal orientations: [100], [110], and [111]. As for the work function of DWCNT, there is a large difference between the GGA and the LDA. At the moment, we cannot establish the reason for the same; however, we discuss the results obtained by GGA since it generally offers higher accuracy. The work function becomes smaller as Pd adsorbs the hydrogen atom. However, the work function of DWCNT is qualitatively higher than that of PdH_x_, and it is not appropriate to consider the charge transfer. Furthermore, if we suppose that the electron gets transferred from PdH_x_ to the CNT, the charge balance is not maintained. Therefore, we consider that electrons released from hydrogen are occupied by Pd; subsequently, the binding between Pd and CNT slightly strengthens as the electrons are partially shared. This might result in the canceling of the holes in the CNT and electrons become the main conductive carriers.

Referring to the result of the Seebeck effect, the main conductive carriers switched from holes to electrons. This is definitely due to the adsorption and dissociative reaction of hydrogen in the palladium nanoparticles. Considering the charge balance, it is reasonable to consider that the electrons are localized in the PdH_x_ nanoparticles and are shared at the Pd/CNT interface rather than being transferred to the CNT.

## 4. Conclusions

In this study, we synthesized a Pd/CNT film and observed the electric resistance changes to develop a future hydrogen sensor. It was found that the higher the temperature, the faster is the dissociation reaction of hydrogen and the response speed, and lower is the maximum amount of change, because the hydrogen storage amount decreased. Unlike previous related studies, owing to the dissociative adsorption of hydrogen, the electric resistance of the p-type CNT thin film decreased, which should originally have increased. When we measured the potential difference induced by the Seebeck effect, it was found that the CNT changed from p-type to n-type because the experiment was conducted under vacuum conditions. Finally, first-principle calculations were performed by ATK to calculate the work functions of Pd, PdHx, and DWCNT, which revealed that the work function became lower on adsorption of hydrogen, as suggested in a previous report. However, from the perspective of charge neutrality, it is appropriate to assume that the electrons are not transferred to the CNT, but localized at the Pd/CNT interface. In any case, there is no doubt that the atoms derived from hydrogen increase or decrease the number of conduction carriers inside the CNT.

## Figures and Tables

**Table 1:** Sample conditions.

Sample Number	2.13-1	2.13-2
First layer		
Materials	MWCNT	
Resistance Ω	840	654
Second layer		
Materials	Pd	
Sputtering power	6 W	
Gas/flow rate	Ar/20 sccm	
Sputtering time	3 min	1 min

**Table 2:** Work functions calculated by ATK-VNL.

	GGA-PBE(FHI-DZP)			LDA-PZ(FHI-SZP)		
Orientation	[100]	[110]	[111]	[100]	[110]	[111]
Pd	5.18	4.91	5.29	5.60	5.38	5.78
PdH	5.90	5.56	5.37	5.71	5.64	5.37
DWCNT (6, 6)-(12, 12)	5.22			6.48		

The unit of all numbers (energies) is eV.

## References

[1] Dunn S. (2002). Hydrogen futures: Toward a sustainable energy system. Int. J. Hydrog. Energy.

[2] Rowsell J.L., Yaghi O.M. (2005). Strategies for hydrogen storage in metal-organic frameworks. Angew. Chem. Int. Ed..

[3] Eberle U., Felderhoff M., Schueth F. (2009). Chemical and physical solutions for hydrogen storage. Angew. Chem. Int. Ed..

[4] Hübert T., Boon-Brett L., Buttner W. (2015). Sensors for Safety and Process Control in Hydrogen Technologies.

[5] Agbossou K., Chahine R., Hamelin J., Laurencelle F., Anouar A., St-Arnaud J.M., Bose T.K. (2001). Renewable energy systems based on hydrogen for remote applications. J. Power Sources.

[6] Tabib-Azar M., Sutapun B., Petrick R., Kazemi A. (1999). highly sensitive hydrogen sensors using palladium coated fiber optics with exposed cores and evanescent field interaction. Sens. Actuators B.

[7] Flanagan T.B., Oates W.A. (1991). The palladium-hydrogen system. Annu. Rev. Mater. Sci..

[8] Frazier G.A., Glosser R.J. (1980). Characterization of thin films of the palladium-hydrogen system. J. Less Common Met..

[9] Lewis F.A. (1967). The palladium Hydrogen System.

[10] Maksymovych N.P., Oleksenko L.P., Nikitina N.V. (2020). Semiconductor materials Ce-SnO2/Sb2O5 and Pd-SnO2/Sb2O5 for creating sensitive elements of sensors for hydrogen. Theor. Exp. Chem..

[11] Cabrera A.L., Aguayo-Soto R. (1997). Hydrogen absorption in palladium films sensed by changes in their resistivity. Catal. Lett..

[12] Lee E., Lee J.M., Koo J.H., Lee W., Lee T. (2010). Hysteresis behavior of electrical resistance in Pd thin films during the process of absorption and desorption of hydrogen gas. Int. J. Hydrog. Energy.

[13] Kim K.R., Noh J.S., Lee J.M., Lee W. (2010). Suppression of phase transitions in Pd thin films by insertion of a Ti buffer layer. J. Mater. Sci..

[14] Im Y., Lee C., Vasquez R.P., Bangar M.A., Myung N.V., Menke E.J., Penner R.M. (2006). Investigation of a single Pd nanowire for use as a hydrogen sensor. Small.

[15] Walter E.C., Favier F., Penner R.M. (2002). Palladium mesowire arrays for fast hydrogen sensors and hydrogen-actuated switches. Anal. Chem..

[16] Atashbar M.Z., Banerji D., Singamaneni S. (2005). Room-temperature hydrogen sensor based on palladium nanowires. IEEE Sens. J..

[17] Jeon K.J., Lee J.M., Lee E., Lee W. (2009). Individual Pd nanowire hydrogen sensors fabricated by electron-beam lithography. Nanotechnology.

[18] Kim K.T., Sim J., Cho S.M. (2006). Hydrogen gas sensor using Pd nanowires electro-deposited into anodized alumina template. IEEE Sens. J..

[19] Kong J., Chapline M.G., Dai H. (2001). Functionalized carbon nanotubes for molecular hydrogen sensors. Adv. Mater..

[20] Joshi R.K., Krishnan S., Yoshimura M., Kumar A. (2009). Pd nanoparticles and thin films for room temperature hydrogen sensor. Nanoscale Res. Lett..

[21] Ju S., Lee J.M., Jung Y., Lee E., Lee W., Kim S.J. (2010). Highly sensitive hydrogen gas sensors using single-walled carbon nanotubes grafted with Pd nanoparticles. Sens. Actuators B.

[22] Gurlo A., Clarke D.R. (2011). High-sensitivity hydrogen detection: Hydrogen-induced swelling of multiple cracked palladium films on compliant substrates. Angew. Chem..

[23] Kim J.H., Jeon J.G., Ovalle-Robles R., Kang J.T. (2018). Aerogel sheet of carbon nanotubes decorated with palladium nanoparticles for hydrogen gas sensing. Int. J. Hydrog. Energy.

[24] Erünal E., Ulusal F. (2020). Thermal effects on hydrogen storage capacity of Pd/MWCNT nanoparticles deposited at moderate pressure and temperature supercritical carbon dioxide conditions. J. Supercrit. Fluids.

[25] Lee J., Shim W., Lee E., Noh J., Lee W. (2011). Highly mobile palladium thin films on an elastomeric substrate: Nanogap-based hydrogen gas sensors. Angew. Chem..

[26] Hu Y., Perello D., Mushtaq U., Yun M. (2008). A single palladium nanowire via electrophoresis deposition used as an ultrasensitive hydrogen sensor. IEEE Trans. Nanotechnol..

[27] Perdew J.P., Burke K., Ernzerhof M. (1996). Generalized gradient approximation made simple. Phys. Rev. Lett..

[28] Perdew J.P., Burke K., Ernzerhof M. (1997). Generalized gradient approximation made simple (vol 77, pg 3865, 1996). Phys. Rev. Lett..

[29] Perdew J.P., Zunger A. (1981). Self-Interaction Correction to Density-Functional Approximations for Many-Electron Systems. Phys. Rev. B.

[30] Kokabu T., Takashima K., Inoue S., Matsumura Y., Yamamoto T. (2017). Transport phenomena of electrons at the carbon nanotube interface with molecular adsorption. J. Appl. Phys..

[31] Inoue S., Kokabu T., Matsumura Y. (2018). Effects of physical and chemical adsorption on the electric conductance of carbon nanotube films. AIP Adv..

[32] Inoue S., Tomita Y., Kokabu T., Matsumura Y. (2018). Principles of detection mechanism for adsorbed gases using carbon nanotube nanomat. Chem. Phys. Lett..

[33] Gupta C.K. (2003). Chemical Metallurgy: Principles and Practice.

[34] Kokabu T., Inoue S., Matsumura Y. (2016). Estimation of adsorption energy for water molecules on a multi-walled carbon nanotube thin film by measuring electric resistance. AIP Adv..

[35] Schlapbach L., Züttel A. (2001). Hydrogen-storage materials for mobile applications. Nature.

[36] Han M., Jung D., Lee G.S. (2014). Palladium-nanoparticle-coated carbon nanotube gas sensor. Chem. Phys. Lett..

